# High-Efficient Liquid Exfoliation of Boron Nitride Nanosheets Using Aqueous Solution of Alkanolamine

**DOI:** 10.1186/s11671-017-2366-4

**Published:** 2017-11-17

**Authors:** Bangwen Zhang, Qian Wu, Huitao Yu, Chaoke Bulin, He Sun, Ruihong Li, Xin Ge, Ruiguang Xing

**Affiliations:** 1 School of Materials and Metallurgy, Inner Mongolia University of Science and Technology, Baotou, 014010 China; 2 Instrumental Analysis Center, Inner Mongolia University of Science and Technology, Baotou, 014010 China

**Keywords:** BN nanosheets, Monoethanolamine, Liquid exfoliation, Yield, Composite

## Abstract

As one of the simple and efficient routes to access two-dimensional materials, liquid exfoliation has received considerable interest in recent years. Here, we reported on high-efficient liquid exfoliation of hexagonal boron nitride nanosheets (BNNSs) using monoethanolamine (MEA) aqueous solution. The resulting BNNSs were evaluated in terms of the yield and structure characterizations. The results show that the MEA solution can exfoliate BNNSs more efficiently than the currently known solvents and a high yield up to 42% is obtained by ultrasonic exfoliation in MEA-30 wt% H_2_O solution. Finally, the BNNS-filled epoxy resin with enhanced performance was demonstrated.

**Electronic supplementary material:**

The online version of this article (10.1186/s11671-017-2366-4) contains supplementary material, which is available to authorized users.

## Background

Since the discovery of graphene in 2004, the interests in graphene and its analogues two-dimensional materials [1, 2] are ever growing throughout the world. Single-layer or few-layer boron nitride nanosheets (BNNSs), known as “white graphene,” share near identical structure to graphene that the sp^2^ hybridized B and N atoms covalently bind into hexagonal crystal in single layers, resulting in weak van der Waals forces between them. Due to the structure and a wide band gap (5.5 eV) [2], BNNSs are endowed with outstanding mechanical, thermal, and dielectric properties, as well as excellent chemical stability, thus exhibiting great potentials in applications such as transparent films [3, 4], protective coating [5, 6], advanced composites [7–9], dielectrics [10, 11], and electronic devices [12, 13] etc.

To produce ultra-thin BNNSs, a variety of methods such as ball milling [14–16], intercalation-oxidation [17, 18], chemical vapor deposition (CVD) [3, 4], and liquid exfoliation [2, 19–28] have been developed. Of these methods, both ball milling and intercalation-oxidation methods are time-consuming and prone to induce impurity and defects in samples, while the CVD costs highly and is applied to prepare continuous film instead of disperse nanosheets that are more popular in practical applications. Recently, liquid exfoliation of BNNSs from hexagonal boron nitride (hBN) powder has received much attention because it is easy to use, economical, free of defect, etc. The driving forces were ascribed dynamically to sonic vibration [19, 20] or liquid shear [21, 22], and thermodynamically to minimization of Gibbs mixing free energy [23, 24] or interfacial energy [25] between the nanosheets and solvents. According to the latter, the composition and properties of used solvent play an important role in liquid exfoliation. Much research has shown that hBN can be exfoliated preferentially in few pure solvents such as N-methyl-2-pyrrolidone (NMP), dimethylformanmide (DMF), and isopropanol (IPA) [9, 19–24, 26] and some mixed solvents [25, 27–29]. However, high-efficient and cheap solvents for hBN liquid exfoliation have been rarely reported, limiting the large-scale preparation and applications of BNNSs.

In the present paper, monoethanolamine (MEA) aqueous solution was attempted for the first time for liquid exfoliation of BNNSs. It was found to exfoliate hBN more efficiently than the other solvents with very high yield. Moreover, this solution has higher specific surface tension (SST) than that of known solvents. The obtained BNNSs were characterized by X-ray diffraction (XRD), scanning electron microscopy (SEM), transmission electron microscopy (TEM), Raman, and X-ray photoelectron spectroscopy (XPS) techniques. Finally, as an example, the BNNSs were employed to reinforce epoxy resin (ER). The obtained composites exhibited improved thermal and mechanical properties.

## Results and Discussion

**Fig. 1:**
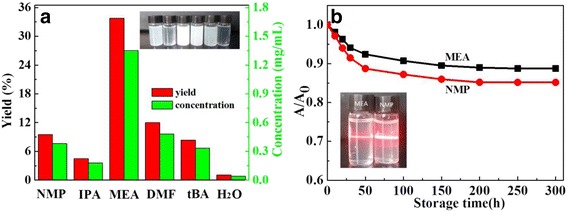
Comparison of **a** the yield and suspension concentration of BNNSs exfoliated in various solvents and **b** normalized absorbance of the MEA- and NMP-exfoliated suspensions vs. storage time

Figure 1a compares six solvents with respect to the yield and suspension concentration of exfoliated BNNSs. It is obvious that among these solvents, MEA earns the highest yield up to 33.7%. The resulting suspension is milk-white (see the inset) with a high concentration of 1.3 mg/mL. In contrast, the yield of other solvents is 12% (DMF), 9.5% (NMP), 8.4% (tBA), 4.5% (IPA), and 1.5% (H_2_O), far below that of the former. The suspensions produced from these solvents are transparent or semi-transparent because of noticeable precipitation. To evaluate the stability of exfoliated dispersion, we measured the UV-vis absorbance (400 nm) normalized to its initial value (*A*/*A*_0_) of the MEA-exfoliated suspension dependent on storage time, as shown in Fig. 1b. For comparison, the absorbance of NMP-exfoliated suspension was given together. It shows both two suspensions are stable, so the *A*/*A*_0_ retains about 90 and 86% respectively after standing for 50 h, when Tyndall scattering is still clear, as shown in the inset of Fig. 1b.

**Fig. 2:**
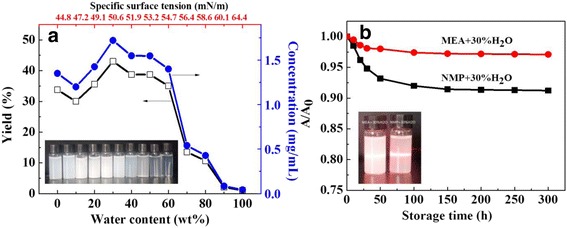
**a** Dependence of the yield and suspension concentration of BNNSs exfoliated in MEA aqueous solution on mass percents of water and the corresponding specific surface tension of the solution. **b** Normalized absorbance of MEA-30 wt% H_2_O- and NMP-30 wt% H_2_O-exfoliated suspensions vs. storage time

To further investigate the exfoliation in MEA aqueous solution, the yield and suspension concentration of exfoliated BNNSs dependent on mass percent of water with the corresponding specific surface tension (SST) were plotted in Fig. 2a. With the increase of water content, the yield first decreases and then increases to a peak value, followed by a drop in succession till near zero in pure water. The highest yield of 42% that corresponds to the suspension concentration of 1.5 mg/mL was achieved in MEA-30 wt% H_2_O solution with a high SST more than 50 mJ/m^2^. To our best knowledge, this yield is possibly the highest value reported in literatures for liquid exfoliation of BNNSs and other two-dimensional materials (Additional file 1: Table S1). Even compared to other exfoliation methods (Additional file 1: Table S2), such as intercalation and ball milling exfoliation, this result is still highly competitive. Also, the MEA solution can keep a higher exfoliation yield (more than 30%) when its water content varies widely from 20 to 60 wt%, implying that BNNSs can be prepared more economically in this solution than other pure solvents. Similarly, we compared the stability of two suspensions exfoliated by MEA-30 wt% H_2_O and NMP-30 wt% H_2_O respectively in Fig. 2b. As compared to Fig. 1b, they show higher concentration and increased absorbance due to the increased yield, the corresponding absorbance increases by 8 to 98% and by 5 to 91%. This indicates the stability of exfoliated product increases due to the addition of water. From now on, we specify the BNNSs as those exfoliated by MEA-30 wt% H_2_O.

**Fig. 3:**
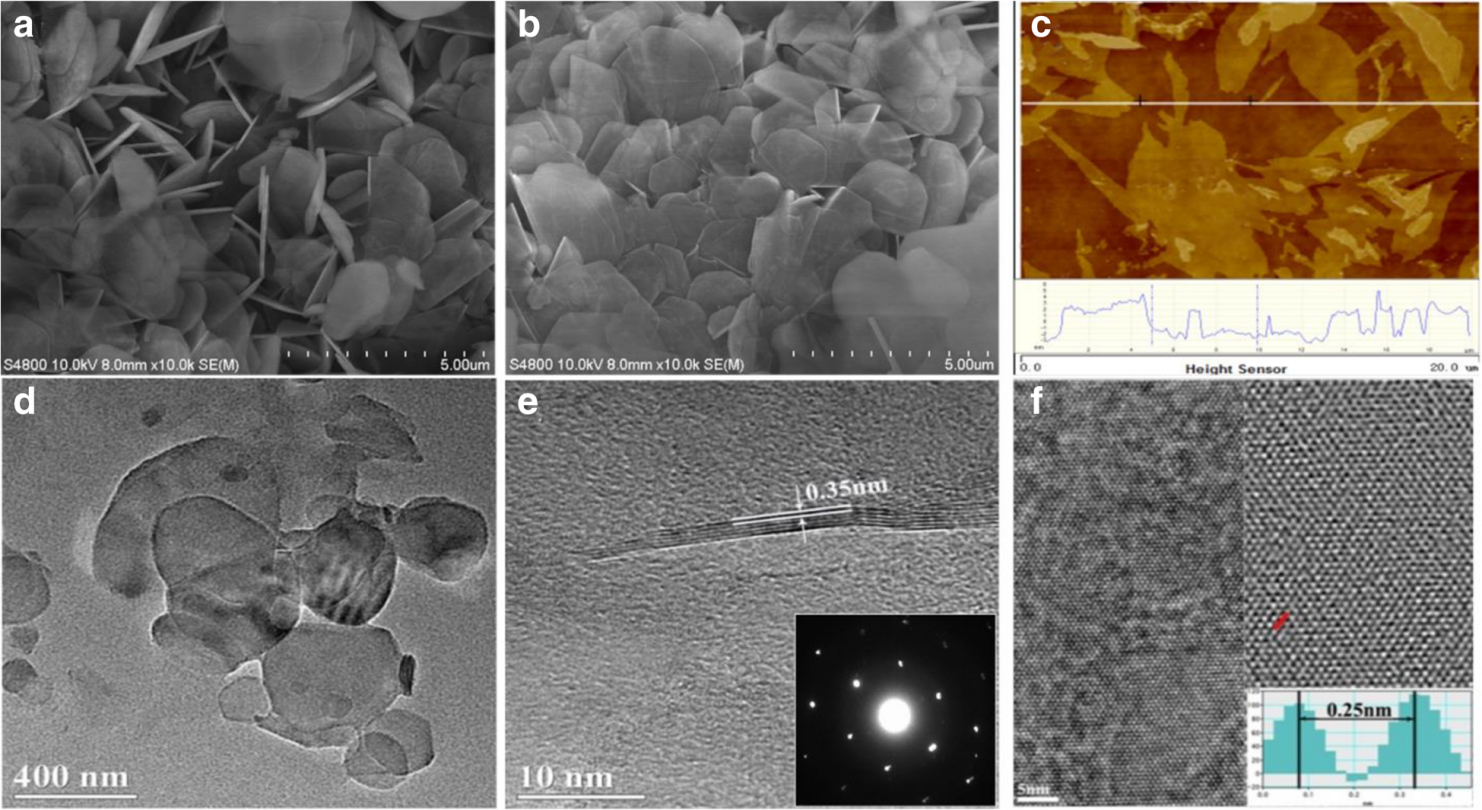
SEM images of pristine hBN (**a**) and BNNSs (**b**), AFM image (**c**), TEM images (**d**–**e**) of BNNSs, and high-resolution TEM image (**f**) of a BNNS (left) and its IFFT image (right)

Now two problems arise from above observations: first, how to understand the superior exfoliation performance of MEA compared with other solvents; second, why can the introduction of water in appropriate amount in MEA improve the exfoliation? Regarding the first problem, we resort to the solubility parameter theories (SPTs). Following these theories, Coleman et al. [23, 24] suggested that the solvents for effective exfoliation were those with dispersive, polar, and H-bonding solubility parameters matching those of layered materials in order to minimize the exfoliation energy. They found that hBN was most effectively dispersed in those solvents with a SST close to 40 mJ/m^2^. In other studies [16, 28], this value was reported as 20~40 mJ/m^2^. In our system, pure MEA has a SST of 44.8 mJ/m^2^ according to ref. [30] (where the data at 50 °C was taken according to the experimental condition, the same below), which roughly agrees with this case. However, when MEA is mixed with 20~60 wt% of water (the second problem), an enhanced exfoliation was observed in this solution, whose SST is about 49~55 mJ/m^2^ (Fig. 2a) and much higher than the previous values. This enhanced exfoliation which occurred in high SST of mixed solvents is presumably due to following factors: (1) MEA molecules that tend to form a network or ring-like structure due to the interactions among amino and hydroxyl groups are disaggregated by the added water molecules [31], allowing them to intercalate BN layers more easily and enhance the exfoliation; (2) the water makes the amino groups of MEA absorbed on BNNSs hydrolyzed and increases surface potential of the BNNSs, hence introducing an additional electrostatic stability, as observed in MEA-30 wt% H_2_O-exfoliated BNNS suspension (Fig. 2b); (3) more or less addition of the water would deviate from the condition above and restrict the liquid exfoliation.

Figure 3a, b displays the SEM images of pristine hBN and BNNSs, respectively. Pristine hBN exhibits as two-dimensional self-standing platelets with lateral dimension of about 0.5~5 μm and initial thicknesses more than 100 nm. In contrast, owing to effective exfoliation, BNNSs lie flat on the substrate and the top layers are transparent to electron beams to see the bottom layers (Fig. 3b). The atomic force microscopy (AFM) image (Fig. 3c) shows most of the exfoliated BNNSs are less than 5 nm in thickness. The morphology of these BNNSs was further characterized by TEM (Fig. 3d–f). As observed in Fig. 3d, several very thin BNNSs cover the supporting film, whose morphology is similar to the SEM image. Figure 3e demonstrates an exfoliated five-layer-atom BNNS with thickness of about 1.8 nm. Its interplanar spacing is measured as 0.35 nm, corresponding to the (002) plane. The selected area electron diffraction (inset in Fig. 3e) reveals the good sixfold symmetry of BNNSs, indicating that BNNSs are structurally integral and not damaged during ultrasonic exfoliation. High-resolution TEM image (Fig. 3f, left) together with its inverse fast Fourier transform (IFFT) (Fig. 3f, right) confirms the hexagonal atom configuration of BNNSs, and the inset in IFFT image indicates that the center distance between adjacent hexagonal rings is 0.25 nm [14].

**Fig. 4:**
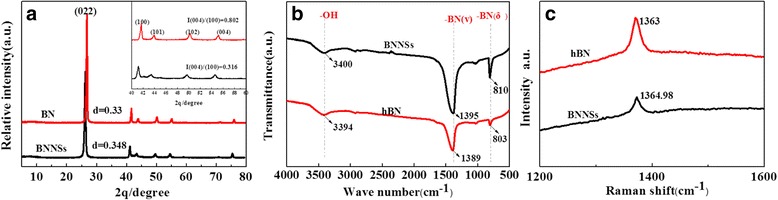
**a** XRD, **b** FTIR, and **c** Raman analysis of pristine hBN and BNNSs

Figure 4a gives the XRD pattern of pristine hBN and BNNSs. The hexagonal phase of pristine hBN is characterized by the peaks at 2*θ* = 26.8°, 41.7°, 43.9°, 50.2°, and 55.2°, which correspond to the (002) (*d*_002_ = 0.33 nm), (100), (101), (102), and (004) planes, respectively. In contrast, these peaks of BNNSs show a reduced intensity and sharpness, which correlated with their weakened c-direction stacking [29]. A slight shift of (002) peak from 2*θ* = 26.8° (hBN) to 26.2° (BNNSs) indicates an increased layer interspacing (*d*_002_ = 0.35 nm). Furthermore, the inset reveals that the intensity ratio of (004) peak to (100) peak, *I*_004_/*I*_100_, of BNNSs is 0.316, far less than that of hBN (0.802), which can be interpreted by the preferred orientation of exfoliated (004) or (002) plane [26]. Figure 4b shows the Fourier transformation infrared spectroscopy (FTIR) spectra of pristine hBN and BNNSs. The hBN has two characteristic peaks at 1389 and 803 cm^−1^, presenting the in-plane B–N stretching and out-of-plane B–N bending vibration, respectively. They blue shift to 1395 and 810 cm^−1^ when the hBN was exfoliated into BNNSs. This shift can be attributed to the thinning of hBN after exfoliation, which enhances the stretching vibration and specially bending vibration of B–N bonds. In addition, the weaker band at ~3400 cm^−1^ is correlated with O–H or N–H stretching vibrations or absorbed water molecules, widely observed in BN materials. The Raman spectra were presented in Fig. 4c. The strong peak at 1366.8 cm^−1^ presents the high-frequency interlayer Raman active E_2g_ mode of pristine hBN. Upon exfoliation, it red shifts to 1363.8 cm^−1^ with an increased full width at half maximum, implying the reduced interlayer interaction of exfoliated products [32, 33], which agrees with the XRD and FTIR results.

**Fig. 5:**
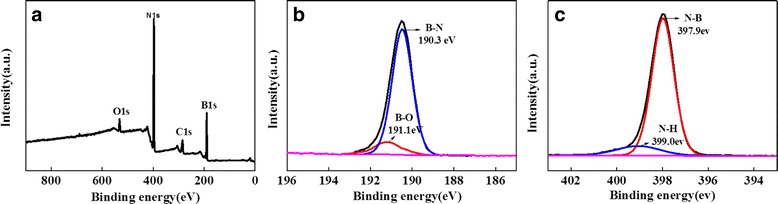
XPS spectra of **a** BNNS survey, **b** B1s, and **c** N1s

The chemical and bond composition of BNNSs was further characterized by XPS (Fig. 5). XPS survey (Fig. 5a) shows the coexistence of B and N as main elements and O and C as impurities in the samples. The B/N ratio is about 1.07, close to the reported values [16, 34]. In the B1s spectrum (Fig. 5b), the peak can be fitted using two components: B–N bond (190.4 eV) and B–O bond (191.2 eV). The latter may be formed due to the hydrolysis [35] of B atoms in defected BN layers or the adsorbed H_2_O into BN layers during exfoliation. In the N1s spectrum (Fig. 5c), the small peak in 399 eV is assigned to N–H bond, which is possibly introduced together in hydrolysis. The hBN exhibits similar XPS spectra (Additional file 1: Figure S1) except that there is a slightly reduced B–O bond contribution in B1s peak and absence of N–H bond contribution in N1s peak.

**Fig. 6:**
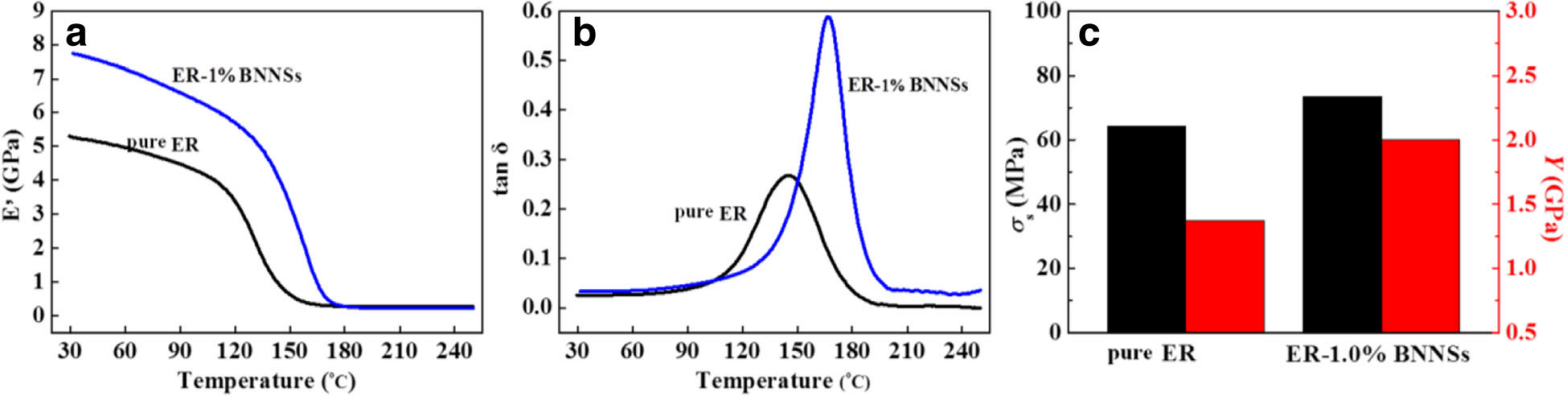
**a** Storage modulus *E*′, **b** loss modulus tan *σ*, and **c** tensile strength *σ*_s_ and Young’s modulus *Y* of pure ER and ER-1% BNNSs

As an application, we prepared ER-BNNS composite by dispersing the BNNSs in ER polymer. Figure 6 conducts the DMA (a, b) and mechanical tests (c) for the obtained ER-1% BNNS composite and pure ER. Figure 6a shows that the storage modulus *E*′ of the composite is higher than pure ER in the glassy state, indicating enhanced rigidity of the composite. This can be ascribed to the introduction of rigid PVP-functionalized BNNSs, as well as the strong interaction between the BNNSs and ER matrix. Figure 6b gives the dependence of loss modulus tan*σ* of pure ER and ER-1% BNNSs on temperature, where the temperature corresponding to loss peak indicates glass transition temperature *T*_g_ [36]. It shows pure ER has a *T*_g_ peak at 130 °C with an intensity of 0.28. After addition of 1 wt% PVP-functionalized BNNSs (BNNSs-PVP) in ER, the *T*_g_ peak shifts to 165 °C with increased intensity of 0.58. This suggests that two-dimensional BNNSs can, on the one hand, effectively restrict the segmental motion and relaxation of ER polymers by space limit and interface bonding so that the *T*_g_ of composite increases and, on the other hand, create numerous heterointerfaces throughout the matrix, leading to the increases of stress loss. To evaluate the reinforcement effect of the BNNSs, Fig. 6c compares tensile strength *σ*_s_ and Young’s modulus *Y* for pure ER and ER-1% BNNSs. It shows *σ*_s_ = 64.25 MPa and *Y* = 1.3 GPa for pure ER, while *σ*_s_ = 73.5 MPa and *Y* = 2.01 GPa for ER-1% BNNSs. That is, an addition of only 1 wt% BNNSs-PVP increases *σ*_s_ and *Y* of the ER by 14.4 and 53.8%, respectively. The property comparison suggests that our ER-1% BNNS composite is superior to most BN-filled polymers reported in literatures (Additional file 1: Table S3).

## Conclusions

In summary, we reported on MEA aqueous solution as a new type of mixed solvents for high-efficient and cost-effective liquid exfoliation of BNNSs. The control experiments show MEA can exfoliate hBN superior than currently known solvents, and this ability can be further improved by the addition of water of appropriate amount in MEA. In the optimum, an exfoliation yield more than 40% was achieved in MEA-30 wt% H_2_O solution. Also, we found that this solution, when resulting in the most efficient exfoliation of BNNSs, has a much high SST which deviated greatly from the predictions by SPTs, suggesting that additional interactions might need to be considered in SPTs to better interpret the liquid exfoliation. The exfoliated BNNSs demonstrate an ability of significantly improving the thermal and mechanical properties of polymers. The mixed solvent here reported enables the scalable exfoliation and applications of BNNSs and exhibits great potentials in other exfoliation techniques, such as shear exfoliation and ball milling exfoliation, and other two-dimensional materials.

## Methods

### Materials

hBN powder (1~5 μm, 99.5%), monoethanolamine (MEA), N-methyl-2-pyrrolidone (NMP), isopropanol (IPA), dimethylformanmide (DMF), tert-butanol (tBA), polyvinylpyrrolidone (PVP, molecular weight ~8000), methylhexahydrophthalicanhydride (MeHHPA), and 2,4,6-tris(dimethyl -aminomethyl) phenol (DMP-30), purchased from Aladdin industrial corporation of Shanghai, were of reagent grade. Bisphenol-A epoxy resin (epoxide number 0.48~0.54) was provide by Baling Company, SINOPEC.

### Preparation of BNNSs

Typically, 200 mg of pristine hBN powders was mixed in 50 mL of MEA or MEA aqueous solution with given water content in a 200-mL beaker, before sonicated for 4 h at about 50 °C in a 6-L bath sonicator (KQ3200DA, Kunshan Shumei) operating at 40 kHz and supplied power dissipation of 150 W. The resultant suspension was centrifugated at 3500 rpm for 20 min. The supernatant was decanted to afford a concentrated solution of exfoliated BNNSs. It was washed with ethanol repeatedly and vacuum dried at 100 °C overnight, giving the BNNS powder. The yield is defined as the mass ratio of exfoliated BNNSs to pristine hBN. For comparison, several popular solvents such as NMP, DMF, IPA, and tBA were chosen to exfoliate hBN powders following the same process.

### Preparation of ER-BNNS Composite

First, 30 mg BNNS powder and 100 mg PVP were dispersed in 10 mL of DMF. Then the dispersion was stirred at 100 °C for 6 h, allowing PVP to adhere to the surfaces of BNNSs. The resultant suspension was separated, washed, and dried following the above procedure, giving PVP-functionalized BNNSs (BNNSs-PVP). Third, bisphenol-A epoxy resin, MeHHPA, and BNNSs-PVP (41:57.5:1 by mass ratio) were mixed for 40 min before being vacuum degassed at 60 °C for 20 min; the mixture was added with 0.5% DMP-30 as the promoter and then sonicated for 10 min. Finally, the resulting paste was casted in a mold and cured using a heating procedure: 80 °C/10 h + 100 °C/3 h + 150 °C/3 h, forming the ER-1% BNNS composite sheets as testing samples. For comparison, pure ER samples were obtained using the above process in the absence of BNNSs-PVP. The samples are tape-like (DMA test) with a size 10 mm × 25 mm × 1 mm or dumbbell-like (mechanical test) with thickness of 1 mm.

### Characterization

Optical absorption spectra were taken from a spectrophotometer (UV-vis; Persee T1910). The chemical components were analyzed using Fourier transformation infrared spectroscopy (FTIR; Bruker IFS66V), Raman spectrometry (RS; HORIBA JY, LabRAMXploRA ONE), and X-ray photoelectron spectroscopy (XPS; Kratos Axis Supra, Al-Kα radiation). The phases were identified by X-ray diffraction (XRD; PANalytical, X’Pert PRO, Cu-Kα radiation, 1.54 Å). The morphology and size of nanosheets were observed using field-emission scanning electron microscopy (SEM; Hitachi, S4800), transmission electron microscopy (TEM; JEOL, JEM-2010), and atomic force microscopy (AFM; Bruke, Dimension Icon). For ER/BNNS samples, dynamic mechanical analysis was carried out with a dynamic mechanical analyzer (DMA; DMA8000, Perkin Elmer) based on a single cantilever mode at a frequency of 1 Hz. Tensile strength and Young’s modulus were measured using an electronic universal testing machine (CMT-200, Jinan Liangong) with a load range of 0~200 kN.

## Additional file

Additional file 1: Table S1. Liquid exfoliation comparison of BNNSs and other two-dimensional materials. **Table S2.** Comparison of BNNSs exfoliated by various methods. **Table S3.** Property comparison of various BN/polymer composites. (DOC 2240 kb)
