# Supplementary material for: High-Efficient Liquid Exfoliation of Boron Nitride Nanosheets Using Aqueous Solution of Alkanolamine
Source: Nanoscale Res Lett. 2017 Nov 17;12:596. doi: 10.1186/s11671-017-2366-4 (PMC5691823; doi:10.1186/s11671-017-2366-4)
Supplement: Additional file 1: Table S1. — Liquid exfoliation comparison of BNNSs and other two-dimensional materials. Table S2. Comparison of BNNSs exfoliated by various methods. Table S3. Property comparison of various BN/polymer composites. (DOC 2240 kb) [file 11671_2017_2366_MOESM1_ESM.doc]

Supplementary

High-efficient liquid exfoliation of boron nitride nanosheets using aqueous solution of alkanolamine

Bangwen Zhang1, 2 *, Qian Wu1, Huitao Yu1, Chaoke Bulin1, He Sun1, Ruihong Li1, Xin Ge1, Ruiguang Xing1

1 School of Materials and Metallurgy, 2 Instrumental Analysis Center, Inner Mongolia University of Science and Technology, Baotou 014010, China

*Corresponding author 
Ph. D. Bangwen Zhang: bangwenz@126.com 


Figure S1 XPS spectra of hBN (a) survey, (b) B1s and (c) N1s


Table S1 Liquid exfoliation comparison of BNNSs and other two-dimensional materials
Products	Journals	Solvents	SST (mJ/m2)	Exfoliation condition	Yield (%)	Refs.	
BNNSs,
Graphene, MoS2	Science,
2011, 331(4): 568	NMP, IPA, DMF etc.	30~40	SC 24h,
CF 1500rpm /45min	<5%	1	
BNNSs
Graphene, MoS2	Angew. Chem,
2011, 50, 10839	EtOH+H2O	25~30	SC 8h,
CF 3000rpm/20min	<5%	2	
BNNSs	J. Phys. Chem. C
2011, 115: 2679	H2O	-	SC 8h,
CF 3000rpm	low	3	
BNNSs	Nanos Res Lett
2013, 8:49	IPA	23	SC 20h,
CF 14000/10min	low	4	
BNNSs	Mater. Expr
2014, 4: 165	IPA + ammonia solution	-	SC 20h,
CF 3000rpm	5-18%	5	
BNNSs	Chem. Commun.,
2015,51, 187	(EtOH, IPA, tBA
etc) +H2O	20~30	SC 3h,
CF 3200/20min	-	6	
BNNSs	Chem. Commun., 2015, 51: 12068	Ionic liquids	32-50	SC 8h,
CF 3000/20min	1.6-36%	7	
BNNSs	ACS Appl. Mater. Interf, 2016, 8: 9881	SOCl 2	33.3	SC 20h,
CF 2000-2500/5min	20%	8	
BNNSs	ACS Appl. Mater. Interf, 2016, 8: 27064	Chlorosulfonic acid + H2O	-	SC 16h,
CF 3000/10min	19.1%	9	
BNNSs,
Graphene, MoS2	Nature comm,
2015, 6: 8294	H2O	-	SC 60h,
CF <1000rpm/30min	18%	10	
BNNSs		MEA+H2O	45~55	SC 4h,
CF 3500rpm/20min	42%	This work	
Graphene	Nature nanotech,
2008, 3: 563	NMP, DMF, IPA etc	35-45	SC 30min
CF 500rpm/90min	low	11	
Graphene,
MoS2	Nature comm,
2013, 4: 2213	(MET,ETA,IPA,TBA)+H2O	25-30	SC 3h,
CF 1450rpm/20min	low	12	
Graphene	Carbon,
2015, 95: 802	NMP	40	SC 2.5h,
CF 1500rpm/45min	< 3%	13	
Black Phosphorus	Chem. Commun.,
2014, 50: 13338	NMP	40	SC 24-48h,
CF 1500rpm/45min	-	14	
Black Phosphorus	Adv. Mater.
2015,27:1887	DMF, DMSO	36.5-44	SC 15h,
CF 2000/30min	low	15	
MoS2	Chem. Mater.
2016, 28:337	NMP+H2O	-	SC 1h, CF 2000-12700rpm/10min	-	16	
WS2	ACS Nano
2016, 10: 1589	H2O+ surfactant	-	SC 1h
CF 1500-10000rpm/2h,	-	17	
       
Note: SC (sonication), CF (centrifugation), SST (specific surface tension) of the solvents applied when resulting in the most efficient exfoliation


Table S2 Comparison of BNNSs exfoliated by various methods
Methods	Products	Journals	Solvents or aids	Exfoliation condition	Yield (%)	Refs.	
Intercalation exfoliation	S-doped BNNSs	Nanoscale,
2014, 6: 11671	KMnO4, H3PO4, H2SO4	Intercalation 75 °C /12h,
CF 6000rpm/30min	25%	18	
Hydrothermal exfoliation	F-doped BNNSs	Angew. Chem,
2014, 126: 3719	H2O +NH4F	24h	4.4%	19	
Thermal Exfoliation	Oxidized BNNSs	Small
2014, 10: 2352	Air	Heating 1000 °C >1h,
SC	65%	20	
Thermal Exfoliation	HO-modified
BNNSs	Adv. Mater.,
2015, 27: 7196	H2O gas	Heating 850 °C >2h,
CF 1200 rpm/30 min	-	21	
Ball-mining	Defected- BNNSs	Nano Lett. ,
2015, 15: 1238	NMP, DMF, IPA, EtOH etc.	BM 24h,
CF 2000 rpm/30min	18%	22	
Ball milling
exfoliation	Defected- BNNSs	Nature Comm,
2015, 6: 8849	urea	BM 20h, without CF	85%	23	
Liquid exfoliation	BNNSs		MEA+H2O	SC 4h,
CF 3500rpm/20min	42%	This work	
          
                  Note: CF (centrifugation), BM (ball-milling)


Table S3 Property comparison of various BN/polymer composites
polymers	Fillers	Tg increment
(ºC)	ós increment
 (MPa)	Y increment
(GPa)	Refs.	
ER	70wt% BN microparticles	<25	-	-	24	
ER	1wt% BN nanoflake	-	8.5	0.3	25	
ER	5wt% BNNSs	10-14	-	-	26	
PC	0.1wt% BNNSs, 	-	15.6	0.35	27	
PI	10wt% BNNSs	4	-	-	28	
BECY	5vol% BN nanoparticles	-4	-	-	29	
PMMA	14wt% BN nanotubes	13	-	-	30	
PMMA	0.3wt% BNNSs	2.3	5	0.39	31	
ER	1wt% BNNSs	35	8.3	0.71	This work	
                    
 Note: Tg (glass transition temperature), ós (tensile strength), Y (Young's modulus)

Reference
[1] Coleman, J. N.; Lotya, M.; O'Neill, A.; Bergin, S. D.; et al. Two-dimensional nanosheets produced by liquid exfoliation of layered materials. Science, 2011, 331, 568-571.
[2] Zhou, K. G.; Mao, N. N.; Wang, H. X.; Peng, Y.; Zhang, H. L. A Mixed-Solvent Strategy for Efficient Exfoliation of Inorganic Graphene Analogues. Angew. Chem. Int. Ed., 2011, 50, 10839-10842.
[3] Lin, Y.; Williams, T. V.; Xu, T.; Cao, W., et al. Aqueous Dispersions of Few-Layered and Monolayered Hexagonal Boron Nitride Nanosheets from Sonication-Assisted Hydrolysis: Critical Role of Water, J. Phys. Chem. C 2011, 115, 2679-2685.
[4] Xue, Y.; Liu, Q.; He, G.; Xu, K.; Jiang, L. Hu, X.; Hu, J. Excellent electrical conductivity of the exfoliated and fluorinated hexagonal boron nitride nanosheets. Nanos. Res. Lett . 2013, 8, 49.
[5] Cao, L.; Emami, S.; Lafdi, K. Large-scale exfoliation of hexagonal boron nitride nanosheets in liquid phase. Mater. Express. 2014, 4, 165-171.
[6] Marsh, K. L.; Soulimana, M.; Kaner, R. B. Co-solvent exfoliation and suspension of hexagonal boron nitride. Chem. Comm. 2015, 51, 187-190.
[7] T Morishita, H Okamoto, Y Katagiri, M Matsushita and K Fukumori. A high-yield ionic liquid-promoted synthesis of boron nitride nanosheets by direct exfoliation, Chem. Commun., 2015, 51: 12068-12071
[8] W Sun, Y Meng, Q Fu, F Wang, G Wang, W Gao, X Huang,and F Lu. High-Yield Production of Boron Nitride Nanosheets and Its Uses as a Catalyst Support for Hydrogenation of Nitroaromatics, ACS Appl. Mater. Interf 2016, 8: 9881−9888
[9] T Morishita, and H Okamoto. Facile Exfoliation and Noncovalent Superacid Functionalization of Boron Nitride Nanosheets and Their Use for Highly Thermally Conductive and Electrically Insulating Polymer Nanocomposites, ACS Appl. Mater. Interf 2016, 8: 27064−27073 
[10] Kim, J.; Kwon, S.; Cho, D.; Kang, B., et al. Direct exfoliation and dispersion of two-dimensional materials in pure water via temperature control. Nature Comm. 2015, 6, 8294.
[11] Hernandez, Y.; Nicolosi, V.; Lotya, M.; Blighe, F. M., et al. High-yield production of graphene by liquid-phase exfoliation of graphite, Nature Nanotech, 2008, 3: 563-568.
[12] Halim, U.; Zheng, C. R.; Chen, Y.; Lin, Z., et al. A rational design of cosolvent exfoliation of layered materials by directly probing liquid–solid interaction. Nature Comm, 2013, 4, 2213.
[13] Arao, Y.; Kubouchi, M. High-rate production of few-layer graphene by high-power probe sonication. Carbon, 2015, 95: 802-808.
[14] Brent, J .R.; Savjani, N.; Lewis, E. A.; Haigh, S. J., et al. Production of few-layer phosphorene by liquid exfoliation of black phosphorus. Chem. Comm., 2014, 50, 13338-13341.
[15] Yasaei, P.; Kumar, B.; Foroozan, T.; Wang, C., et al. High-Quality Black Phosphorus Atomic Layers by Liquid-Phase Exfoliation. Adv. Mater. 2015, 27,1887-1892.
[16] Jawaid, A .; Nepal, D.; Park, K,; Jespersen, M., et al. Mechanism for Liquid Phase Exfoliation of MoS2. Chem. Mater. 2016, 28, 337-348.
[17] Backes, C.; Szyd³owska, B. M.; Harvey, A.; Yuan, S., et al. Production of Highly Monolayer Enriched Dispersions of Liquid-Exfoliated Nanosheets by Liquid Cascade Centrifugation. ACS Nano 2016, 10, 1589-1601.
[18] Bhimanapati,G. R.; Kozuchab, D.; Robinson, J. A. Large-scale synthesis and functionalization of hexagonal boron nitride nanosheets. Nanoscale 2014, 6, 11671-11675.
[19] Du, M.; Li, X.; Wang, A.; Wu, Y., et al. One-Step Exfoliation and Fluorination of Boron Nitride Nanosheets and a Study of Their Magnetic Properties. Angew. Chem. 2014, 126, 3719 -3723.
[20] Cui, Z.; Oyer, A. J.; Glover, A. J.; Schniepp, H. C.; Adamson, D. H. Large Scale Thermal Exfoliation and Functionalization of Boron Nitride. Small, 2014, 10, 2352-2355.
[21] Xiao, F.; Naficy, S.; Casillas, G.; Khan, M. H., et al. Edge-Hydroxylated Boron Nitride Nanosheets as an Effective Additive to Improve the Thermal Response of Hydrogels. Adv. Mater. 2015, 27, 7196-7203.
[22] Lee, D.; Lee, B.; Park, K. H.; Ryu, H. J., et al. Scalable Exfoliation Process for Highly Soluble Boron Nitride Nanoplatelets by Hydroxide-Assisted Ball Milling. Nano Lett. 2015, 15, 1238-1244.
[23] Lei, W.; Mochalin, V. N.; Liu, D.; Qin, S., et al. Boron nitride colloidal solutions, ultralight aerogels and freestanding membranes through one-step exfoliation and functionalization. Nature Comm, 2015, 6: 8849.
[24] K Kim, M Kim, Y Hwang, J Kim. Chemically modified boron nitride-epoxy terminated dimethylsiloxane composite for improving the thermal conductivity, Ceramics Intern, 2014, 40 : 2047
[25] D Lee, S H Song, J Hwang, S H Jin, K H Park, B H Kim, S H Hong, and S Jeon. Enhanced Mechanical Properties of Epoxy Nanocomposites by Mixing Noncovalently Functionalized Boron Nitride Nanoflakes, Small, 2013, 9: 2602–2610
[26] J Yu, X Huang, C Wu, X Wu, G Wang, P Jiang. Interfacial modification of boron nitride nanoplatelets for epoxy composites with improved thermal properties, Polymer, 2012, 53: 471-480
[27] T Sainsbury, A Satti, P May, A O'Neill, V Nicolosi, Y K Gun'ko, and J N Coleman. Covalently Functionalized Hexagonal Boron Nitride Nanosheets via Nitrene Addition, Chem. Eur. J., 2012, 18: 10808-10812.
[28] M Tsai, I Tseng, J Chiang, and J Li. Flexible Polyimide Films Hybrid with Functionalized Boron Nitride and Graphene Oxide Simultaneously To Improve Thermal Conduction and Dimensional Stability,  ACS Appl. Mater. Interf, 2014, 6: 8639−8645
[29] H Wu and M R Kessler. Multifunctional Cyanate Ester Nanocomposites Reinforced by Hexagonal Boron Nitride after Noncovalent Biomimetic Functionalization, ACS Appl. Mater. Interf, 2015, 7: 5915−5926
[30] X Wang, C Zhi, Q Weng, Y Bando and D Golberg. Boron Nitride Nanosheets: novel Syntheses and Applications in polymeric Composites,J Physics: Conference Series, 2013,471: 012003
[31] C Zhi, Y Bando, C Tang, H Kuwahara, and D Golberg. Large-Scale Fabrication of Boron Nitride Nanosheets and Their Utilization in Polymeric Composites with Improved Thermal and Mechanical Properties, Adv. Mater. 2009, 21: 2889–2893
